# Lack of P2Y_13_ in mice fed a high cholesterol diet results in decreased hepatic cholesterol content, biliary lipid secretion and reverse cholesterol transport

**DOI:** 10.1186/1743-7075-10-67

**Published:** 2013-11-06

**Authors:** Laeticia Lichtenstein, Nizar Serhan, Wijtske Annema, Guillaume Combes, Bernard Robaye, Jean-Marie Boeynaems, Bertrand Perret, Uwe J F Tietge, Muriel Laffargue, Laurent O Martinez

**Affiliations:** 1 INSERM, UMR 1048, Institut des Maladies Métaboliques et Cardiovasculaires, Toulouse 31432, France; 2 Université de Toulouse III, UMR 1048, Toulouse 31300, France; 3 Department of Pediatrics, University of Groningen, University Medical Center Groningen, Groningen, Netherlands; 4 Institute of Interdisciplinary Research, IRIBHM, Université Libre de Bruxelles, Gosselies, Belgium; 5 CHU de Toulouse, Hôpital Purpan, Toulouse, France

**Keywords:** P2Y_13_, HDL, HDL-uptake, High cholesterol diet, Bile lipid secretions, Reverse cholesterol transport, Cholesterol metabolism, Liver, ATP synthase

## Abstract

**Background:**

The protective effect of HDL is mostly attributed to their metabolic function in reverse cholesterol transport (RCT), a process whereby excess cellular cholesterol is taken up from peripheral cells, processed in HDL particles, and later delivered to the liver for further metabolism and biliary secretion. Mechanistically, the purinergic P2Y_13_ ADP-receptor is involved in hepatic HDL endocytosis (i.e., uptake of both HDL protein + lipid moieties), which is considered an important step of RCT. Accordingly, chow-fed P2Y_13_ knockout (P2Y_13_^-/-^) mice exhibit lower hepatic HDL uptake, which translates into a decrease of hepatic free cholesterol content and biliary cholesterol and phospholipid secretion.

**Findings:**

The aim of this study was to determine the effect of high cholesterol diet (HCD) in P2Y_13_^-/-^ mice, in order to mimic high dietary cholesterol intake, which is a major cause of dyslipidemia in humans. As previously reported with chow-diet, HCD did not affect plasma lipid levels in P2Y_13_^-/-^ compared with control mice but decreased hepatic free and esterified cholesterol content (p < 0.05, P2Y_13_^-/-^ versus control). Interestingly, biliary lipid secretion and macrophages-to-feces RCT were more dramatically impaired in P2Y_13_^-/-^ mice fed a HCD than chow-diet. HCD did not enhance atherosclerosis in P2Y_13_^-/-^ compared with control mice.

**Conclusion:**

This study demonstrates that high dietary cholesterol intake accentuated the metabolic phenotype of P2Y_13_^-/-^ mice, with impaired hepatobiliary RCT. Although other animal models might be required to further evaluate the role of P2Y_13_ receptor in atherosclerosis, P2Y_13_ appears a promising target for therapeutic intervention aiming to stimulate RCT, particularly in individuals with lipid-rich diet.

## Findings

### Introduction/research hypothesis

Dyslipidemia, reflected by either high triglyceride or cholesterol plasma concentrations, is a major risk factor of atherosclerosis [1]. The risk of atherosclerosis, a leading cause of cardiovascular disease and death, is inversely correlated to plasma high-density lipoprotein cholesterol (HDL-C). The protective effect of HDL particles is mostly attributed to their central function in Reverse Cholesterol Transport (RCT), a process whereby peripheral excessive cholesterol, especially that contained in macrophage foam cells, is taken up to be processed in HDL particles, and later delivered to the liver for final excretion into the feces either as neutral sterols or after metabolic conversion into bile acids [2]. This process, which represents a major pathway of the body to eliminate proatherogenic cholesterol, relies on specific interactions between HDL particles and cells, both peripheral (cholesterol efflux) and hepatic cells (cholesterol output). We recently identified a new pathway for holoparticle HDL endocytosis by the liver (i.e., hepatic uptake of both HDL protein + lipid moieties), involved in RCT. In this pathway, apoA-I, the major protein of HDL, binds an ecto-F_1_-ATPase leading to ATP hydrolysis into ADP [3]. Extracellular ADP activates the purinergic P2Y_13_ ADP-receptor, which stimulates *in fine* HDL uptake through an unknown low affinity receptor, distinct from the classical HDL receptor, SR-BI. Our recent work has confirmed the role of the P2Y_13_ receptor in HDL-mediated RCT *in vivo *[4]*.* We showed that P2Y_13_-deficient mice (P2Y_13_^-/-^) exhibited a decrease in hepatic HDL uptake, hepatic cholesterol content, and biliary cholesterol output, although their plasma HDL-C and other lipid levels were normal. These metabolic changes translated into a substantial decrease in the rate of macrophage-to-feces RCT. Therefore, key features of RCT were impaired in P2Y_13_^-/-^ mice.

In order to investigate the role of P2Y_13_ in a dyslipidemic context, we studied the phenotype of P2Y_13_^-/-^ mice fed high cholesterol diet (HCD) for 16 weeks. Our results show that chronically increased cholesterol intake accentuates the metabolic phenotype of P2Y_13_^-/-^ mice, with impaired hepatobiliary metabolism. Specifically, (i) hepatic HDL uptake mediated by P2Y_13_ receptor plays an important role in regulating liver cholesterol content, (ii) P2Y_13_ receptor is essential for normal biliary lipid secretion and fecal excretion of cholesterol originating from macrophages, (iii) these effects of P2Y_13_ activity on the flux of HDL toward the liver does not affect HDL-C level per se or selected HDL functions. Overall, this work emphasizes the essential role of P2Y_13_ in RCT in a dyslipidemic context.

## Materials and methods

### Animals and diets

The animals were caged in an animal facility with alternating 12 h periods of light (07:00 am-7:00 pm) and dark (7:00 pm-07:00 am). 8 week-old male P2Y_13_^-/-^ and P2Y_13_^+/+^ littermates mice (C57BL/6 background) were fed for 16 weeks a high cholesterol diet (Harlan TD 96335, 1.25% cholesterol) then used for experimentation. All animal procedures were in accordance with the guidelines of the Committee on Animals of the Midi-Pyrénées Ethics Committee on Animal Experimentation and with the French Ministry of Agriculture license.

### Plasma lipoprotein analyses

Plasma samples were collected at 11 am, after a fasting period of 3 h. Total cholesterol and triglycerides were measured with commercial kits (CHOD-PAP for cholesterol and GPO-PAP for triglycerides; BIOLABO SA, Maizy, France). Quantification of plasma lipoproteins was performed using an Ultimate® 3000 HPLC system (Dionex, USA) as previously described [5].

### Hepatic lipid analyses

Hepatic cholesterol and triglycerides were analyzed, following Bligh & Dyer lipid extraction, by gas–liquid chromatography, as previously described [4].

### Cannulation of the common bile duct and bile lipid analysis

Mice were fasted for 3 hours and were then anesthetized by intra-peritoneal injection of ketamine hydrochloride and xylazine hydrochloride. At 11 am, gallbladder was cannulated and bile was harvested for 30 minutes, after a stabilization time of 30 minutes. Bile acid, phospholipid and cholesterol analysis was performed as previously reported [5].

### In vivo macrophage-to-feces RCT

RCT assay was performed as previously described [4]. Briefly, thioglycollate-elicited mouse peritoneal macrophages, harvested from C57BL/6(J) donor mice, were loaded for 24 hours with 50 μg/mL acetylated LDL and 5 μCi / ml ^3^H-cholesterol, then injected intraperitoneally in recipient mice (two million dpm/mouse). Blood samples were taken 6, 24 and 48 hours after macrophages injection, feces were collected continuously for 48 hours and livers were harvested 48 hours after macrophages injection and stored at -80°C until lipid extraction and radioactivity counting [4]. All counts were expressed as a percentage of the administered tracer dose.

### Hepatic gene expression

Liver and whole intestine RNA isolation, reverse transcription and real-time quantitative PCR analysis were performed as previously described [5].

### HDL functionality

HDL were isolated from mouse plasma, after precipitation of apoB-containing lipoproteins with polyethylene glycol-6000 [6]. Anti-oxidative property of HDL was assessed by measuring the capacity of HDL to inhibit the oxidation of native LDL as previously described [6,7]. Anti-inflammatory property of HDL was evaluated on human umbilical vein endothelial cells (HUVECs) by measuring MCP-1 gene expression as previously described [6]. Efflux experiments were performed by measuring cholesterol efflux for 5 hours from primary mouse peritoneal macrophages towards either plasma (1%, v/v) or apoB-depleted lipoproteins (2%, v/v), as previously described [6].

### Aortic sinus quantification

The lesions were estimated according to Paigen and collaborators [8] Briefly, each heart was frozen on a cryostat mount with OCT compound (Tissue-Tek), and stored at -80°C. Hearts were cut using a Leica CM3050S cryostat. Fifty sections of 10-μm thickness were prepared from the top of the left ventricle, where the aortic valves were first visible, up to a position in the aorta where the valve cusps were just disappearing from the field. After drying for 1 hour, the sections were stained with oil red O and counterstained with Mayer's hematoxylin. Five sections out of the 50, each separated by 100 μm, were used for specific morphometric evaluation of intimal lesions using a computerized Leica image analysis system, consisting of a Leica DMRE microscope coupled to a video camera and Leica Qwin Imaging software (Leica Ltd, Cambridge, UK). The first and most proximal section to the heart was taken 100 μm distal to the point where the aorta first becomes rounded. Lipid droplets <500 μm^2^ as well as those located in the media were excluded from the measurements. The mean lesion size (expressed in μm^2^) in these 5 sections was used to evaluate the lesion size of each animal. The coded slides were examined blind in two separate analyses by the same examiner and gave consistent results (r = .97).

### Statistical analysis

All results are presented as means ± SEM. Comparisons between groups were made using the Mann–Whitney test for independent samples. Outcomes of p < 0.05 were considered significant. Analyses were performed using GraphPad Prism 6 software.

## Results and discussion

**Figure 1:**
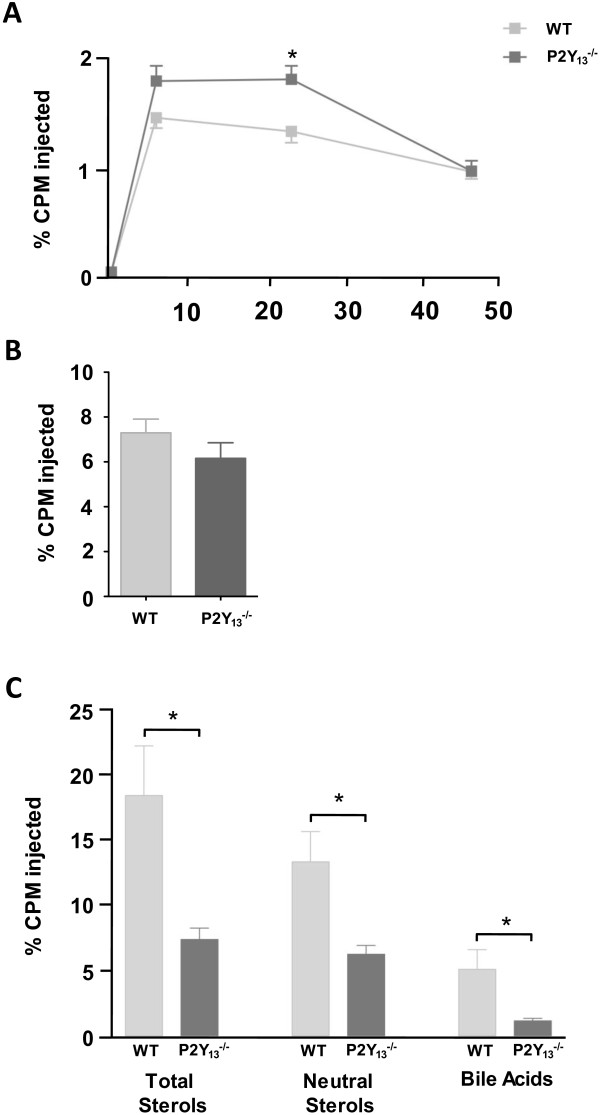
**Lack of P2Y**_**13 **_**in mice fed HCD decreases *in vivo *macrophage-to-feces reverse cholesterol transport.** Two million of ^3^H-cholesterol–labeled peritoneal macrophages from C57BL/6 donor mice were injected intraperitoneally in P2Y_13_^-/-^ (dark grey squares and bars) and WT (light gray squares and bars) mice fed HCD. **(A) **^3^H-cholesterol appearance in plasma 6, 24, and 48 hours after macrophage administration. **(B)**, ^3^H-cholesterol tracer recovery within liver 48 hours after macrophages injection. **(C)**, ^3^H-cholesterol appearance in feces collected continuously from 0 to 48 hours, after macrophages injection. Data are expressed as percent cpm injected ± SEM; n = 6 mice group. Statistically significant differences from WT mice are indicated as *p < 0.05.

In order to investigate the role of P2Y_13_ receptor in a dyslipidemic context, we studied the phenotype of P2Y_13_^-/-^ mice fed high cholesterol diet (HCD) for 16 weeks. Body weight and liver weight were unchanged between P2Y_13_^-/-^ and wild-type (WT, C57BL/6) mice maintained on HCD (data not shown) and plasma total cholesterol, HDL-C, LDL-C and triglycerides did not differ either (Table 1). However, on HCD feeding, hepatic total cholesterol content was significantly lower in P2Y_13_^-/-^ than in WT mice, with decrease in both cholesterol ester and free cholesterol (Table 2). To further assess the effect of HCD on the metabolic phenotype of P2Y_13_^-/-^ mice, we measured biliary flow and lipid secretion, which is considered an essential step in RCT [9]. As reported in Table 3, biliary flow was significantly decreased and biliary secretion of cholesterol, bile acid and phospholipid were also significantly reduced in P2Y_13_^-/-^ as compared to WT mice after 16 weeks of HCD. We next measured the movement of ^3^H-cholesterol from macrophages to the feces, which is a surrogate well-established method to evaluate *in vivo* RCT [10]. We observed that macrophage-to-feces RCT was impaired in P2Y_13_^-/-^ as compared to WT mice, as reflected by a ~60% reduction of total sterol recovered in feces (Figure 1C, -53±5 and -78±4 in % of neutral sterols and bile acids, respectively). This reduced RCT is most likely attributable to the described function of P2Y_13_ receptor in hepatic HDL uptake [4,11]. Accordingly, P2Y_13_^-/-^ mice fed HCD displayed a significant higher ^3^H- tracer present in plasma at 6 and 24 h (Figure 1A) and a trend to lower ^3^H-tracer recovered in the liver at 48 h, as compared to WT mice (Figure 1B). Macrophage-to-feces RCT experiments have frequently reported that ^3^H-tracer recovered in feces is more sensitive to evidence changes in RCT than ^3^H-tracer present in plasma and liver which is often unchanged [10,12], probably because radioactivity recovered in feces represents the endpoint of RCT whereas radioactivity present in plasma and liver depends on cholesterol flux of a freely distributable tracer. This hence might explain the unchanged % of ^3^H-tracer present in plasma at 48 h and the lack of a significance in the decrease of ^3^H-tracer in the liver of P2Y_13_^-/-^ compared to WT mice (Figure 1A, B).

**Table 1:** **Plasma lipid values in P2Y**_**13**_^**+/+ **^**(WT) and P2Y**_**13**_^**-/- **^**mice fed a HCD for 16 weeks**

	**VLDL**		**LDL**		**HDL**	
	**WT**	**P2Y**_**13**_^**-/-**^	**WT**	**P2Y**_**13**_^**-/-**^	**WT**	**P2Y**_**13**_^**-/-**^
TC (mg/dl)	14.1 ± 1.92	12.2 ± 3.42	25.1 ± 1.31	18.5 ± 0.34	85.01 ± 16.9	90.62 ± 8.88
FC (mg/dl)	4.55 ± 0.71	3.31 ± 0.76	10.3 ± 4.57	6.00 ± 0.20	33.58 ± 2.56	29.45 ± 2.80
EC (mg/dl)	9.86 ± 1.48	8.93 ± 2.65	14.8 ± 3.06	12.5 ± 0.29	53.33 ± 14.2	61.49 ± 6.1
TG (mg/dl)	24.6 ± 1.27	23.5 ± 4.52	7.26 ± 1.05	9.43 ± 1.66	6.75 ± 2.23	2.45 ± 0.27

Values are expressed as means ± SEM; n = 4 mice per group.

**Table 2:** **Hepatic lipid values in P2Y**_**13**_^**+/+ **^**(WT) and P2Y**_**13**_^**-/- **^**mice fed a HCD for 16 weeks**

	**WT**	**P2Y**_**13**_^**-/-**^
Total cholesterol (nmol/mg)	67.11 ± 3.84	50.50 ± 2.53*
Free cholesterol (nmol/mg)	13.28 ± 0.96	9.65 ± 0.54*
Esterified cholesterol (nmol/mg)	53.41 ± 3.37	41.97 ± 1.89*
Triglycerides (nmol/mg)	30.69 ± 3.76	29.59 ± 2.16

Values are expressed as means ± SEM; n = 10 mice per group.

*Indicates significant difference (p < 0.05) from control mice.

**Table 3:** **Biliary lipid values in P2Y**_**13**_^**+/+ **^**(WT) and P2Y**_**13**_^**-/- **^**mice fed a HCD for 16 weeks**

	**WT**	**P2Y**_**13**_^**-/-**^
Bile flow (μl/min/100 g BW)	5.58 ± 0.42	4.35 ± 0.26*
Cholesterol secretion (nmol/min/100 g BW)	3.79 ± 0.27	2.71 ± 0.36*
Bile acid secretion (nmol/min/100 g BW)	209.9 ± 16.0	167.1 ± 10.2*
Phospholipid secretion (nmol/min/100 g BW)	15.7 ± 0.2	9.7 ± 0.2*

Values are expressed as means ± SEM; n = 10 mice per group.

*Indicates significant difference (p < 0.05) from control mice.

P2Y_13_ deficiency results in a ~40% decrease of hepatic messenger RNA (mRNA) expression of the heterodimeric ATP-binding transporter Abcg5 and Abcg8 (Table 4), suggesting that the cholesterol derived from HDL that has been internalized through P2Y_13_ receptor might use the Abcg5/g8 dependent pathway for apical biliary cholesterol secretion [13]. Hepatic gene expression of the other transporters controlling bile acid and phospholipid fluxes in the liver, such as biliary phospholipid transport protein (Abcb4/mdr3), bile salt export pump (Abcb11/bsep), basolateral transporter sodium taurocholate cotransporting polypeptide (Ntcp) and organic anion transport polypeptide (Oatp) were unchanged (Table 4). As reported in Tables 4 and 5, there was also no significant change in the expression of gene controlling bile acid production such as hepatic cholesterol 7 alpha-hydroxylase (Cyp7A1), hepatic sterol 27-hydroxylase (Cyp27A1), hepatic sterol 12-alpha-hydroxylase (Cyp8b1) and intestinal Fibroblast growth factor 15 (Fgf15). Furthermore, the reduction of bile acid secretion observed in P2Y_13_^-/-^ mice compared to WT mice was mainly driven by lower bile flow (Table 3) since the concentration of bile acid in bile was not significantly reduced (data not shown) whereas both secretion and concentration of biliary cholesterol and phospholipid were decreased (Table 3 and data not shown). Altogether these data indicate that the effect of P2Y_13_ deficiency on decreasing bile acid secretion into the bile cannot be attributed to a decrease of hepatic bile acids synthesis or apical transport but is more likely driven by the decrease of biliary flow. However, further investigation is required to determine the mechanism by which P2Y_13_-mediated HDL endocytosis regulates biliary lipid secretion process. Indeed, although recent studies evidenced that HDL internalized by hepatocytes are transported to two intracellular pools – a rapid turnover retroendocytic pool involving endosomal recycling compartment (ERC) and a slow-turnover pool, involving multivesicular bodies, that is eventually further transported to lysosomes for degradation [14], the process by which intracellular HDL trafficking governs biliary lipid secretion is still poorly characterized.

**Table 4:** **Effect of HCD in P2Y**_**13**_^**-/- **^**mice on hepatic mRNA expression of genes involved in lipid homeostasis**

**Gene**		**Fold-change**	**Accession N°**	**Gene title**
Scarb1	1.00 ± 0.12	0.86 ± 0.10 (p = 0.67)	NM_016741	Scavenger receptor class B, member 1
Ldlr	1.00 ± 0.17	1.02 ± 0.15 (p = 0.75)	NM_010700	Low density lipoprotein receptor
Abca1	1.00 ± 0.16	0.59 ± 0.05* (p = 0.04)	NM_013454	ATP-binding cassette, sub-family A, member 1
Abcg1	1.00 ± 0.10	0.68 ± 0.07* (p = 0.02)	NM_009593	ATP-binding cassette, sub-family G, member 1
Apoa1	1.00 ± 0.10	0.97 ± 0.07 (p = 0.72)	NM_009692	Apolipoprotein A-I
Cyp7a1	1.00 ± 0.23	1.61 ± 0.24 (p = 0.06)	NM_007824	Cytochrome P450, family 27, subfamily A, polypeptide 1
Cyp27a1	1.00 ± 0.13	1.11 ± 0.13 (p = 0.53)	NM_024264	Cytochrome P450, family 8, subfamily B, polypeptide 1
Cyp8b1	1.00 ± 0.22	1.64 ± 0.26 (p = 0.18)	NM_010012	Cytochrome P450, family 8, subfamily B, polypeptide 1
Abcg5	1.00 ± 0.12	0.64 ± 0.05* (p = 0.03)	NM_031884	ATP-binding cassette, sub-family G, member 5
Abcg8	1.00 ± 0.10	0.61 ± 0.07** (p = 0.003)	NM_026180	ATP-binding cassette, sub-family G, member 8
Abcb4	1.00 ± 0.07	0.74 ± 0.08 (p = 0.06)	NM_008830	ATP-binding cassette, sub-family G, member 4
Abcb11/Bsep	1.00 ± 0.11	1.33 ± 0.10 (p = 0.22)	NM_021022	ATP-binding cassette, sub-family B (MDR/TAP), member 11
Ntcp/Slc10a1	1.00± 0.22	1.51 ± 0.17 (p = 0.11)	NM_011387	Solute carrier family 10 (sodium/bile acid cotransporter family), member 1
Oatp/Slco1a1	1.00 ± 0.16	1.33 ± 0.19 (p = 0.47)	NM_013797	Solute carrier organic anion transporter family, member 1A2
Hmgcr	1.00 ± 0.01	1.02 ± 0.09 (p = 0.44)	NM_008255	3-hydroxy-3-methylglutaryl-Coenzyme A reductase
Srebp2	1.00 ± 0.09	0.74 ± 0.07 (p = 0.06)	NM_033218	Sterol regulatory element binding transcription factor 2

Real-time PCR was performed on individual livers of 3 h fasted mice (n = 10 mice per group). For all genes, the fold change was calculated by dividing the P2Y_13_^-/-^ mice value by the wild-type mice value (e.g. an increase of 80% from wild-type is reported as 1.80). * and ** indicate significant difference (p < 0.05 and p < 0.01 respectively) from wild-type mice.

**Table 5:** **Effect of HCD in P2Y**_**13**_^**-/- **^**mice on intestinal mRNA expression of genes involved in lipid homeostasis**

**Gene**		**Fold-change**	**Accession N°**	**Gene title**
Npc1l1	1.00 ± 0.21	1.39 ± 0.21 (p = 0.36)	NM_207242	Niemann-Pick C1-like protein 1
Abcg5	1.00 ± 0.26	1.53 ± 0.24 (p = 0.36)	NM_026180	ATP-binding cassette, sub-family G, member 5
Abcg8	1.00 ± 0.21	1.47 ± 0.19 (p = 0.18)	NM_026180	ATP-binding cassette, sub-family G, member 8
AbcA1	1.00 ± 0.15	1.52 ± 0.23 (p = 0.18)	NM_013454	ATP-binding cassette, sub-family A, member 1
Abcg1	1.00 ± 0.22	0.89 ± 0.11 (p = 0.94)	NM_009593	ATP-binding cassette, sub-family G, member 1
Scarb1	1.00 ± 0.25	1.41 ± 0.32 (p = 0.73)	NM_016741	Scavenger receptor class B, member 1
Oatp/Slco1a1	1.00 ± 0.41	0.81 ± 0.17 (p = 0.94)	NM_013797	Solute carrier organic anion transporter family, member 1A2
Fgf15	1.00 ± 0.28	1.09 ± 0.27 (p = 0.94)	NM_008003	Fibroblast growth factor 15

Real-time PCR was performed on individual intestine of 3 h fasted mice (n = 6 mice per group). For all genes, the fold change was calculated by dividing the P2Y_13_.^-/-^ mice value by the wild-type mice value (e.g. an increase of 80% from wild-type is reported as 1.80).

Interestingly, hepatic mRNA expression of Abca1 and Abcg1, which contribute to cellular cholesterol efflux towards lipid-poor ApoA-I and HDL particles, respectively [13], was significantly decreased in P2Y_13_^-/-^ mice after 16 weeks of HCD (Table 4). These results might suggest that the decrease of hepatobiliary cholesterol secretion observed in P2Y_13_^-/-^ mice fed a HCD is associated with a decrease of *de novo* HDL formation, thus explaining the unchanged plasma HDL-C levels *per se*, as previously proposed for P2Y_13_^-/-^ mice fed a chow diet [4]. Alternatively, the P2Y_13_-mediated HDL endocytosis pathway might efficiently drive cholesterol from HDL towards biliary lipid secretion but could be quantitatively negligible regarding the steady state concentration of plasma HDL-C. Accordingly, it is now well established that macrophage-derived cholesterol represents only a minor proportion of the total cholesterol transported by HDL particles but is essential to prevent foam cell formation [15] supporting the concept that the dynamics of HDL particles are essential for RCT but not necessary correlated to the static measure of plasma HDL-C concentration.

**Figure 2:**
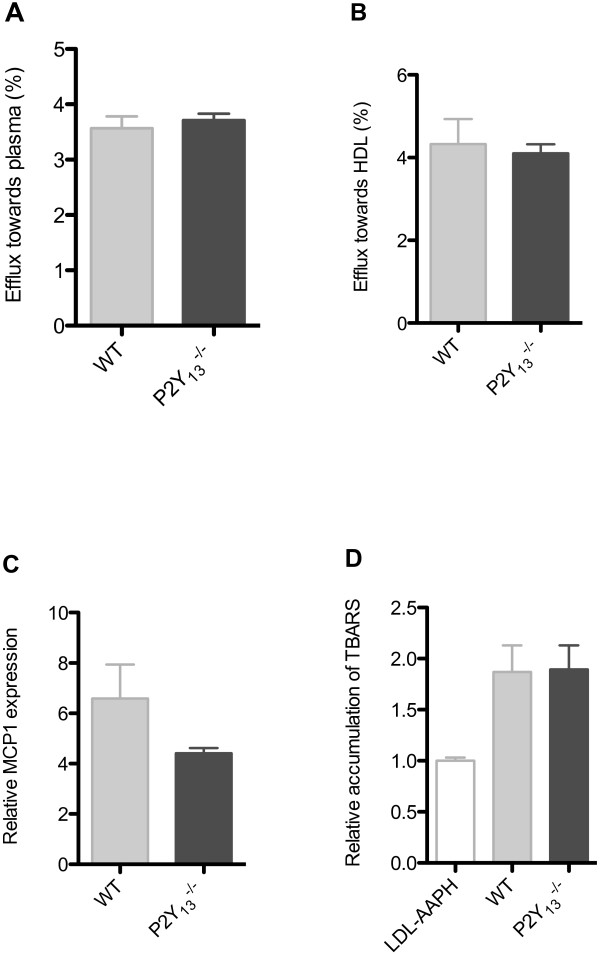
**P2Y**_**13 **_**deficiency does not change the functional properties of HDL on HCD.** HDL function was determined as cholesterol efflux **(A and B)**, protection of HUVECs against inflammation **(C)**, protection of LDL against oxidation **(D)**. Data are presented as means ± SEM, n = 10 mice per group. TBARS: Thiobarbituric aid reactive substances.

**Figure 3:**
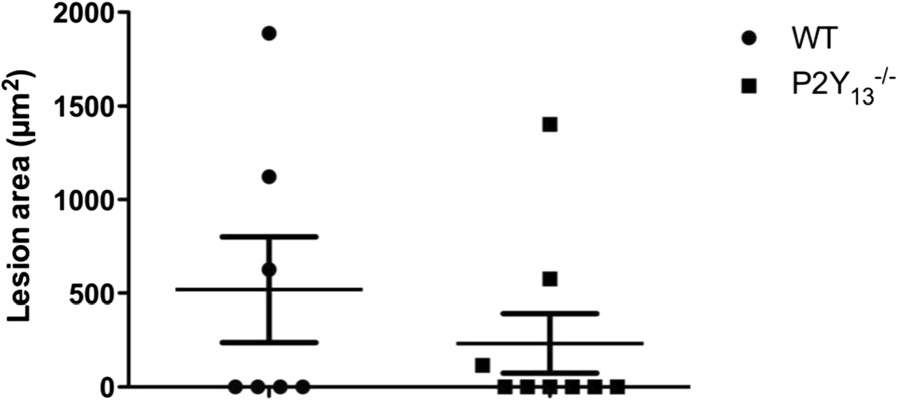
**P2Y**_**13 **_**deficiency does not induce atherosclerosis development on HCD.** Aortic sinus sections were stained with oil red O and lipid content was quantified. Data are presented as means ± SEM, n = 6 in WT group, and n = 9 in P2Y_13_^-/-^ group.

To assess HDL functionality in these mice, we tested whether P2Y_13_ deletion would influence HDL functionalities regarding either their anti-inflammatory or anti-oxidative properties, or their capacity to elicit efflux from cholesterol-loaded macrophages. However, P2Y_13_-deficient mice did not show any change in these respective HDL functionalities after 16 weeks of HCD (Figure 2), suggesting that P2Y_13_ deletion neither interferes with HDL pleiotropic functions, nor HDL-mediated efflux from cholesterol-loaded macrophages. Furthermore, feeding the HCD for 16 weeks did not result in an increased lipid deposition in the aortic sinus of P2Y_13_^-/-^ compared to WT mice (Figure 3). Not surprisingly, these results indicate that P2Y_13_ deletion in C57BL/6 mice does not initiate atherosclerosis development. To formally assess the hypothesis whether P2Y_13_ receptor plays a role in atherosclerosis development, mice lacking P2Y_13_ would have to be crossed with a proatherogenic mouse model.

We previously demonstrated that chow-fed P2Y_13_ deficient mice displayed a reduced uptake of HDL by the liver associated to decreased hepatic free cholesterol content and impaired biliary secretion of cholesterol and phospholipids [4]. These metabolic changes translated into a substantial ~45% decrease in the rate of macrophage-to-feces RCT [4]. In the present study, we observed that a chronic high cholesterol intake accentuates this metabolic phenotype, with a more dramatic impaired hepatobiliary RCT. Indeed, using HCD-fed P2Y_13_^-/-^ compared to WT mice, we reported here that both free and esterified cholesterol content in the liver were decreased (Table 2), together with biliary flow and biliary lipid secretion (Table 3) and a ~60% reduction in the rate of macrophage-to-feces RCT (Figure 1).

P2Y_13_ receptor is also expressed in the intestine [16] and could potentially also regulate intestinal cholesterol absorption or excretion. Our results indicates that the mRNA expression level of the principal genes involved in cholesterol absorption were unchanged between P2Y_13_^-/-^ and WT mice maintained on HCD (Table 5), suggesting that cholesterol absorption is unchanged in P2Y_13_^-/-^ mice.

Overall, our work emphasizes the importance of hepatic P2Y_13_ activity when diet is rich in cholesterol, by regulating biliary lipid output and overall RCT without affecting HDL-C level *per se* or selected HDL functions. Thus, this study opens the way to reconsider pharmacological approaches to target HDL metabolism, particularly with regard to mechanistic aspects of RCT, by improving the flux of circulating HDL towards the liver (e.g., by stimulating P2Y_13_) rather than increasing plasma HDL-C levels.

## Abbreviations

HDL: High density Lipoprotein; LDL: Low density lipoprotein; ApoA-I: apolipoprotein A-I; HCD: High cholesterol diet; RCT: Reverse cholesterol tansport; Ecto-F1ATPase: Ectopic F_1_-ATPase; ATP: Adenosine triphosphate; ADP: Adenosine diphosphate; Abca1: ATP binding cassette subfamily A member 1; Abcb4: ATP binding cassette subfamily B member 4; Abcg1: ATP binding cassette subfamily G member 1; Abcg5: ATP binding Cassette subfamily G member 5; Abcg8: ATP binding Cassette subfamily G member 8; Oatp: Organic anion transport polypeptide; Ntcp: Sodium taurocholate cotransporting polypeptide; WT: Wild-type; EC: Esterified cholesterol; FC: Free cholesterol; TC: Total cholesterol.

## Competing interests

The authors declare that they have no competing of interest.

## Authors’ contributions

LOM conceived the study and participated in its design and coordination. BR and JMB conceived P2Y_13_ knockout mice and participated in the design of the study. LL, NS and GC carried out animal metabolic studies and participated in the design of the study. WA and UJFT carried out HDL functionality assays and analyzed the data. LOM, LL and NS have interpreted the overall data and drafted the manuscript. BP and ML revised the manuscript critically for important intellectual content. All authors read and approved the final manuscript.
